# Temperature mapping on a niobium-coated copper superconducting radio-frequency cavity

**DOI:** 10.1038/s41598-023-44021-w

**Published:** 2023-10-10

**Authors:** Antonio Bianchi, Walter Venturini Delsolaro

**Affiliations:** 1 CERN, Geneva, Switzerland; 2 Present Address: INFN, Milano, Italy

**Keywords:** Applied physics, Techniques and instrumentation

## Abstract

Since the late ’80s, CERN has pioneered the development of niobium thin film radio-frequency (RF) cavities deposited on copper substrates for several particle accelerator applications. However, niobium thin film cavities historically feature a progressive performance degradation as the accelerating field increases. In this study, we describe a temperature mapping system based on contact thermometry, specially designed to obtain temperature maps of niobium-coated copper cavities and, consequently, study the mechanisms responsible for performance degradation. The first temperature maps on a niobium/copper 1.3 GHz cavity are reported along with its RF performance. In addition to some hotspots displayed in the temperature maps, we surprisingly observed a temperature decrease in a limited portion of the cavity cell as the accelerating field increased. This may shed new light on understanding the heat dissipation of niobium thin film cavities in liquid helium-I, which might be exploited to improve the RF cavity performance.

## Introduction

Since the late ‘80s, CERN has pioneered the development of thin film radio-frequency (RF) cavities for particle accelerators. Niobium (Nb) thin film on copper (Cu) cavities have been successfully applied at CERN in the Large Electron Positron collider (LEP-II)^1^, in the Large Hadron Collider (LHC)^2^ and more recently, following the pioneering work done at INFN Legnaro by Palmieri et al.^3^, in the High Intensity and Energy Isotope Separator On Line DEvice (HIE-ISOLDE)^4^. Many efforts are now put in place at CERN in view of their potential implementation in the Future Circular Collider (FCC) machines.

Temperature mapping systems are one of the most valuable diagnostics to investigate mechanisms responsible for performance degradation in superconducting radio-frequency (SRF) cavities. The effectiveness of temperature mapping systems is to detect any loss mechanisms inside the cavity that ultimately produces heat in the cavity substrate^5^. This is also useful to ensure quality control during series production of many SRF cavities. In the last decades, temperature mapping systems significantly contributed to the improvement of cavity performance^5,6^. Indeed, by temperature sensing on the outer surface of SRF cavities, several types of losses that occur inside the cavities can be detected, such as multipacting processes, field emission, ohmic loss mechanisms, and quenches.

One of the first systems for temperature mapping on bulk Nb radio-frequency cavities was built at Cornell University^7^. This system has been duplicated in other laboratories^5,8–11^. All these setups are applied in testing bulk Nb cavities generally operated in superfluid helium (He-II), where the heat exchange from cavities to the helium (He) bath is dominated by the Kapitza resistance. In the past, only one system was developed for Nb thin film cavities deposited on Cu substrates. The system, built at CERN in the ’80s, used a rotating arm of thermometers for mapping Nb/Cu 500 MHz cavities in liquid He above the lambda-point (He-I) in the subcooled condition, precluding crucial insights into how cavities dissipate heat during their standard operating conditions at saturation pressure. Indeed, Nb/Cu cavities are usually operated in He-I at saturation pressure, where the heat transfer from cavities to He bath is different from that in He-I in the subcooled condition as well as in He-II. Another drawback of the system was the long acquisition time, from half an hour to one hour for a complete temperature map. Consequently, only steady-state RF losses in the cavity were detected. In addition, the temperature of the subcooled He bath is not stable over time; in fact, it slowly drifts to higher values. This has to be carefully considered when the acquisition time of a temperature map is quite long.

No temperature mapping systems for Nb/Cu cavities in He-I are currently in operation. This paper describes a system based on contact thermometry, specifically designed for testing Nb/Cu cavities in He-I at saturation pressure and in the subcooled condition. As a result, we can report the first temperature maps of a Nb/Cu 1.3 GHz cavity, along with its RF performance. Temperature mapping of the cavity during its operation showed some interesting sites with characteristics consistent with Joule heating and field emission heating. An optical inspection of the internal cavity surface confirmed the presence of defects on the Nb thin film in correspondence with these sites. Contrary to expectations, we also observed a temperature drop on a limited portion of the cavity cell as the accelerating field increased. This may shed new light on the heat dissipation of Nb/Cu cavities in He-I, and might be exploited to improve the RF cavity performance.

The paper is organized as follows. Section “Temperature mapping on copper surfaces at liquid helium temperatures” gives a brief overview of parameters that play a role in mapping heat losses on Cu surfaces at liquid He temperatures. In section “Temperature mapping system for niobium/copper cavities”, we describe the temperature mapping system developed for testing Nb/Cu 1.3 GHz cavities. After reporting the RF performance of the cavity under test in section “RF performance of the cavity”, we examine the temperature maps acquired at 2.4 K when the He bath is at saturation pressure and in the subcooled condition in section “Temperature maps”. The characterization of heat losses, observed in temperature maps, is presented in section “Characterization of heat losses”, whereas the optical inspection results of the cavity are outlined in section “Optical inspection of the cavity”. Finally, we discuss a potential approach to enhance the heat dissipation of Nb/Cu cavities into the He bath in section “Heat dissipation of niobium-coated copper cavities”.

## Temperature mapping on copper surfaces at liquid helium temperatures

Mapping heat losses on Nb thin film cavities deposited on Cu substrates presents a greater challenge compared to bulk Nb cavities.

**Figure 1:**
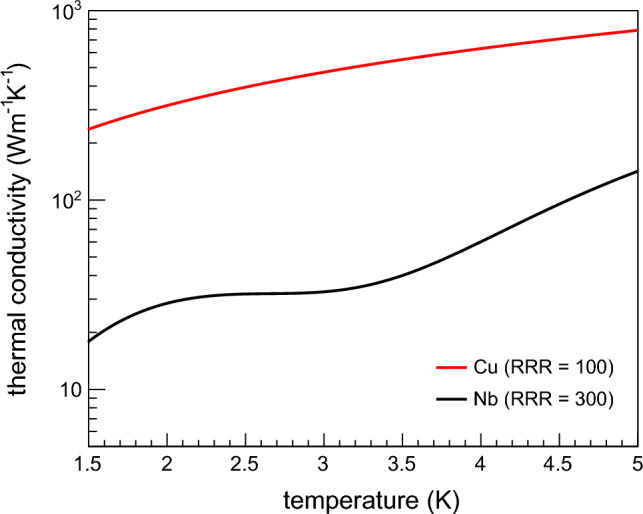
Thermal conductivity of Cu (RRR = 100)^13^ and Nb (RRR = 300)^12^ between 1.5 and 5.0 K.

The thermal conductivity of Cu is more than one order of magnitude higher than that of Nb at liquid He temperatures^12,13^. Figure 1 shows the thermal conductivity of Cu and Nb between 1.5 K and 5.0 K, which is the temperature range where SRF cavities are usually tested. The Cu substrates, generally used for Nb/Cu cavities, have a residual resistance ratio (RRR) between 50 and 100. In contrast, the RRR value of Nb sheets used for manufacturing bulk Nb cavities is usually equal to ~ 300^14^.

**Figure 2:**
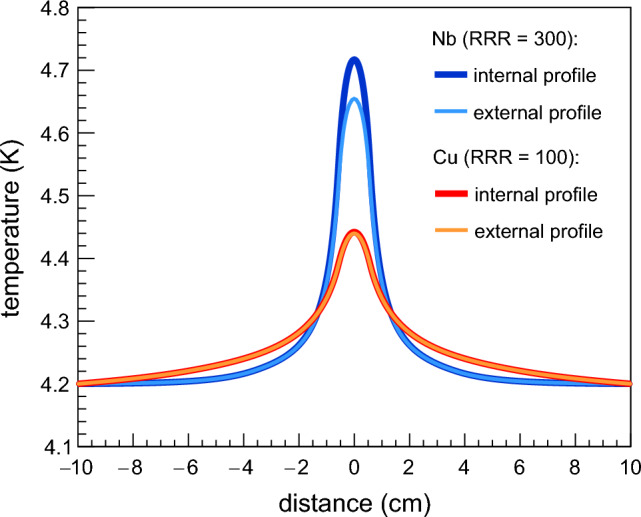
Internal and external temperature profiles in a disk with a diameter of 20 cm and thickness of 2 mm. On the internal side, the disk is heated in its center with a heat flux density of 1.0 W/cm^2^ and cooled by liquid He at 4.2 K at saturation pressure on the external side. The simulated temperature profiles on the Nb (RRR = 300) surface are higher and narrower than those simulated on the Cu (RRR = 100) surface.

The thermal conductivity of Cu is ~ 300 W/(m K) at 1.8 K and ~ 700 W/(m K) at 4.2 K, whereas these values in Nb are only ~ 25 W/(m K) and ~ 60 W/(m K), respectively^12,13^. This implies that the temperature increase on Cu surfaces in correspondence with a given heat loss is lower than that on bulk Nb surfaces. Figure 2 shows the simulated temperature profiles in a disk when a heat loss of 1.0 W/cm^2^ is located at the center. The diameter of the disk is 20 cm and its thickness is equal to 2 mm, which is quite similar to most of the substrates for SRF cavities. The disk is externally cooled by liquid He at 4.2 K at saturation pressure, while it is in a vacuum on the opposite side. The simulation of heat transfer from the disk to He-I is carried out by using data from a prior study conducted at CERN^15^. The simulation is performed using the software COMSOL Multiphysics. In the case of Cu, the internal and external temperature profiles at the center of the disk are lower than those of Nb by more than a factor of 2. In addition, the internal and external temperature profiles on the Cu surface are broader than those on the Nb surface. This makes measuring temperature profiles on Cu surfaces more complex and challenging than on bulk Nb surfaces. At the center of the disk, the difference between the internal and external temperature is ~ 2 mK for Cu, whereas it grows to ~ 60 mK for Nb.

Unlike bulk Nb cavities, Nb/Cu cavities are usually operated in He-I at saturation pressure. In this condition, the heat transfer from the Cu surface to the He bath occurs by convection cooling for low heat fluxes per unit surface area, whereas gaseous He bubbles appear on the Cu surfaces at higher heat fluxes, leading to the transition from convection cooling to nucleate boiling. Cooling by nucleate boiling is more effective than convection cooling in terms of heat transfer from the Cu surface to the He bath^16^. The activation of nucleation sites, where bubbles of gaseous He grow, frequently implies a sudden temperature drop in the surface^16^. According to results of previous studies^17,18^, the ideal condition for mapping heat losses on Nb/Cu cavities in He-I is slightly above the lambda-point of He, which is at ~ 2.17 K. We verified that the He bath temperature of 2.4 K allows us to carry out measurements with satisfactory accuracy. At this temperature, the thermal conductivity of Cu is lower than that at 4.2 K, as shown in Fig. 1. Furthermore, the thermal conductivity of He-I also decreases by ~ 30% from 4.2 to 2.4 K^19^. As a consequence, in the presence of a point-like heat loss, the temperature profiles on Cu surfaces at 2.4 K are higher and broader than those at 4.2 K.

A further parameter to be considered for mapping heat losses on Cu surfaces in He-I is the sensitivity of thermometers. Previous studies show that Allen-Bradley 100 $\Omega$ carbon resistors can be used as thermometers for measuring temperature profiles on Cu surfaces immersed in He-I^17,18^. The sensitivity of Allen-Bradley 100 $\Omega$ resistors at 2.4 K is higher than that at 4.2 K, as will be discussed in Section “Thermometers”.

Another thermodynamic state where heat losses in Nb/Cu cavities can be satisfactorily detected is the subcooled He condition^18^. Indeed, H. Piel in the ’80s observed that the temperature profiles on Cu surfaces in subcooled He-I were much higher and broader than those at saturation pressure^20,21^. This is mainly due to the reduced cooling capability of the He bath in subcooled conditions, which is lower than that at saturation pressure^20,21^. However, Nb/Cu cavities are not generally operated in subcooled He. Therefore, temperature maps of Nb/Cu cavities in He-I at saturation pressure can be used to understand their heat dissipation at operating conditions, whereas temperature mapping in subcooled He allows the localization and characterization of heat losses with high precision and accuracy.

## Temperature mapping system for niobium/copper cavities

A temperature mapping system has been specially developed at CERN to test Nb/Cu cavities. Based on contact thermometry, the system is used to map the temperature on the outer surface of 1.3 GHz single-cell TESLA-type cavities in liquid He-I both at saturation pressure and in the subcooled condition. In the system, temperature sensing is performed using a total of 192 thermometers made of carbon resistors in contact with the outer surface of the cavity. The data acquisition software is developed to provide real-time temperature maps. Therefore, the evolution of heat losses can be monitored and tracked during the cavity cold tests. The design is partially based on that developed at Jefferson Lab for bulk Nb 1.5 GHz CEBAF cavities^5^.

### Thermometers

**Figure 3:**
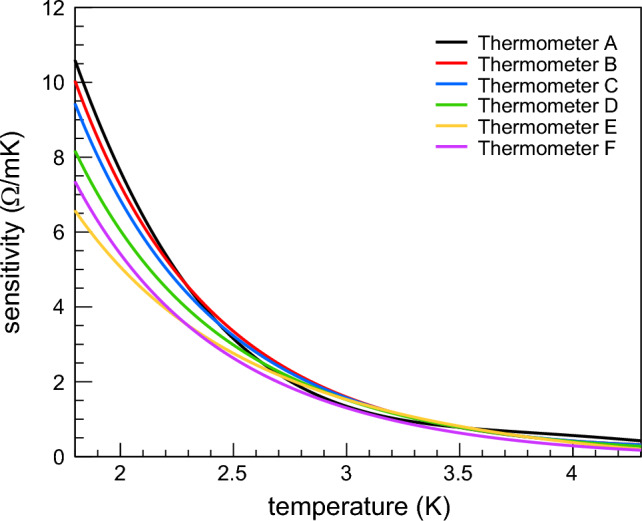
Sensitivity of six Allen-Bradley 100 $\Omega$ carbon resistors between 1.8 and 4.2 K.

For the temperature mapping system, we used 192 Allen-Bradley 100 $\Omega$ carbon resistors as thermometers. The resistance of these carbon resistors increases by decreasing the temperature. Indeed, their resistance is generally ~ 7 k$\Omega$ at 1.8 K, ~ 3 k$\Omega$ at 2.4 K and ~ 1 k$\Omega$ at 4.2 K. Figure 3 shows the sensitivity of six thermometers between 1.8 and 4.2 K.

The resistance variation of Allen-Bradley 100 $\Omega$ carbon resistors is high enough to detect small temperature variations on Cu surfaces in liquid He-I and He-II, as already demonstrated in previous studies^17,18^. However, their resistance slightly changes when a thermal cycle at room temperature is performed. This implies that thermometers must always be calibrated for each cool-down of the system. If no calibration procedure is carried out after each thermal cycle, the uncertainty on temperature can be as high as 150 mK^17^.

Four Temati carbon ceramic thermometers, calibrated from 1.5 to 300 K and immersed in the He bath at the level of the cavity’s equator, are used to calibrate all 192 Allen-Bradley carbon resistors at each cool-down of the system. The response of these calibrated thermometers is acquired by the 4-wire sensing method. In order to calibrate all 192 thermometers of the system, voltage measurements of each thermometer are acquired at ~ 50 mK intervals during the pumping on the He bath, and then correlated to the He bath temperature at the time when each thermometer response was acquired. The calibration curve of each thermometer is obtained by interpolating data with a third-order polynomial function^5^. Once the calibration is completed, the temperature of the cavity measured by all thermometers is acquired without power in the cavity. Then, during the cavity test, the temperature of each thermometer is continuously acquired and subtracted from that measured when no energy was stored in the cavity. This allows the acquisition of real-time temperature maps on the cavity surface during RF tests. The acquisition time of one single temperature map can vary from 35 ms to more than 100 s, depending on the desired accuracy. The typical uncertainty in temperature measurements after calibration is approximately 0.3 mK for an acquisition time of 150 ms for each thermometer.

In the system, each thermometer is embedded in an Accura 25 housing. We verified that Accura 25 3D-printed plastic is suitable for cryogenic temperatures. The housing is sealed by Stycast 2850 FT, which has high thermal conductivity and is impervious to superfluid He. The Accura 25 housing ensures thermal isolation from the He bath, and the Stycast epoxy fully protects them from liquid He. Apiezon N grease is applied between the thermometers and the external cavity surface to lower the thermal contact resistance and, consequently, optimize the temperature measurements^17,18^.

**Figure 4:**
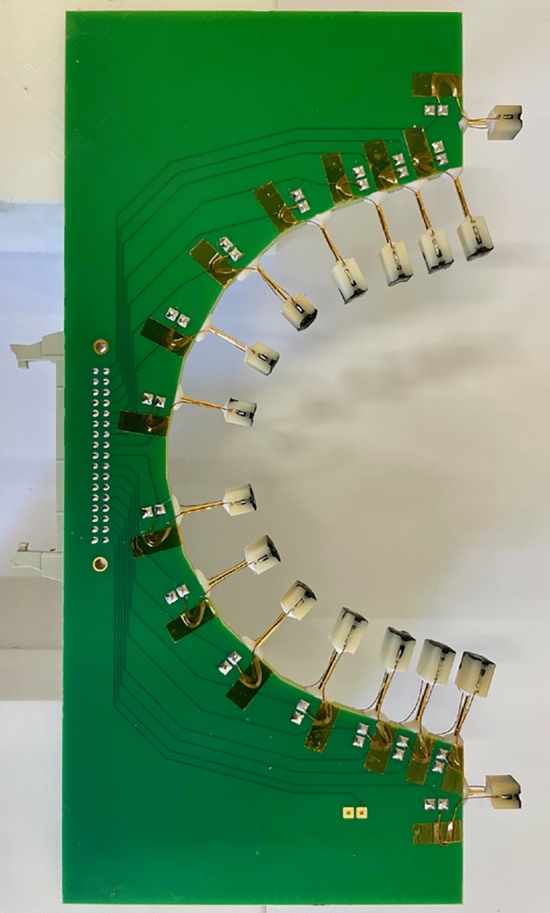
One of the twelve boards used in the CERN temperature mapping system. Each board with sixteen thermometers is machined in order to fit the shape of 1.3 GHz TESLA-type cavities. The thermometers are numbered from 1 to 16 from bottom to top.

Thermometers are placed in contact with the cavity surface by twelve printed circuit boards (PCBs) in FR-4 glass epoxy. Each board with sixteen thermometers is adequately machined to fit the shape of a 1.3 GHz TESLA-type cavity. Each thermometer is pushed towards the cavity by a spring-loaded pin in BeCu. CAF4 silicone rubber is used to glue each spring-loaded pin to the board and the thermometer housing. This material is suitable for cryogenics and can be easily removed when a broken thermometer needs to be replaced. Concerning electrical connections, the wires from each thermometer to the electrical circuit in the PCBs are in manganin, characterized by a low thermal conductivity at liquid He temperatures. Figure 4 shows one of the boards used in the system. The density of thermometers close to the irises of the cavity cell is slightly higher than that at the equator because the temperature profile near the irises is expected to be lower than that at the equator. Hereafter, the thermometers are numbered from 1 to 16 from bottom to top.

**Figure 5:**
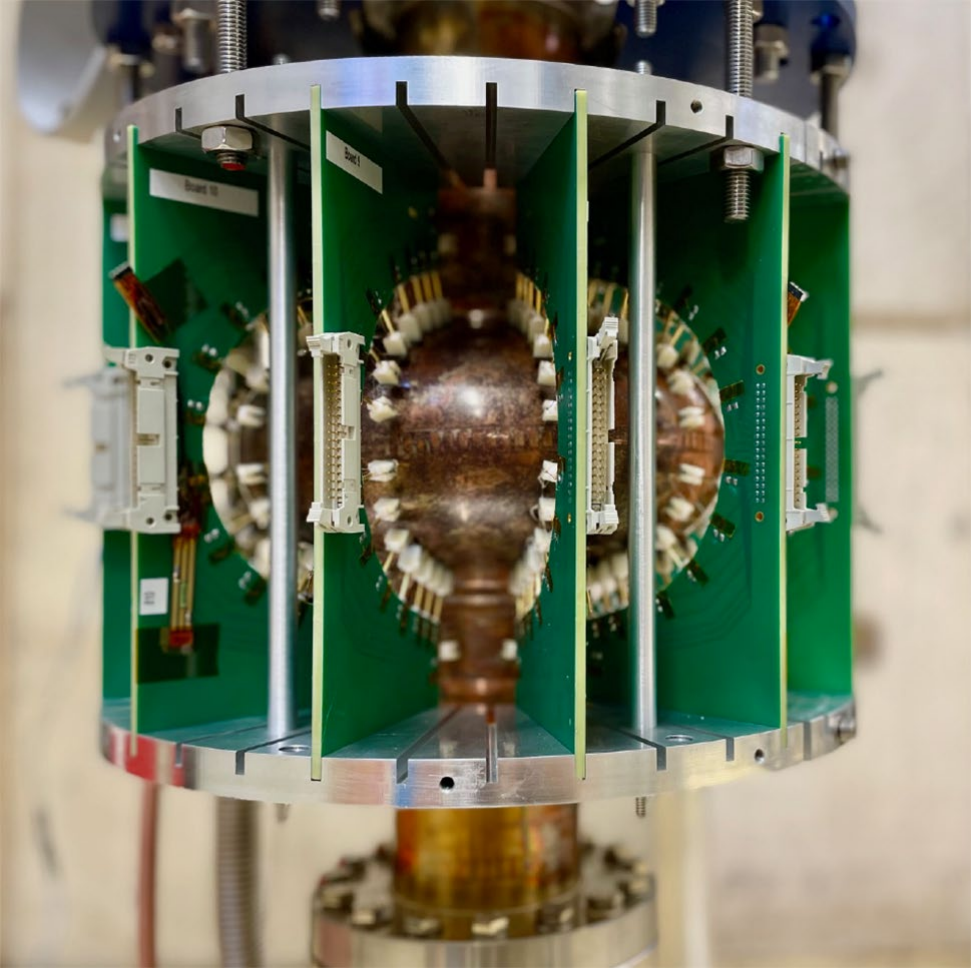
Picture of the temperature mapping system assembled around a Nb/Cu 1.3 GHz single-cell cavity. The twelve thermometer boards are placed azimuthally 30 degrees apart in order to cover uniformly the whole surface of the cavity.

The supporting system, used to place the boards around the cavity under test, consists of four semicircular plates in aluminum. For each ring, 36 radial grooves are machined, where boards can be slided. For assembling the system, four locking rings are bolted on the aluminum plates after sliding the boards in the grooves and, then, the boards are pushed towards the cavity wall by set screws. The supporting system is designed to fit inside the cryostat used for tests, which has a radius of 17 cm. In the standard configuration, the twelve thermometer boards are placed azimuthally 30 degrees apart to cover the cavity uniformly, as shown in Fig. 5. The spacing among the thermometers of neighboring boards varies from ~ 5 cm close to the equator of the cavity cell and ~ 2 cm close to the irises. Using the results of a previous study^18^, the spacing among thermometers is chosen to be enough for detecting heat losses at 2.4 K both at saturation pressure and in subcooled He.

If a limited portion of the cavity needs to be investigated with higher resolution, the boards can be placed with a minimum angle of 10 degrees to increase the density of thermometers. In this configuration, the spacing among thermometers is equal to ~ 18 mm at the equator and ~ 7 mm at the irises of the cavity cell.

### Electronics

**Figure 6:**
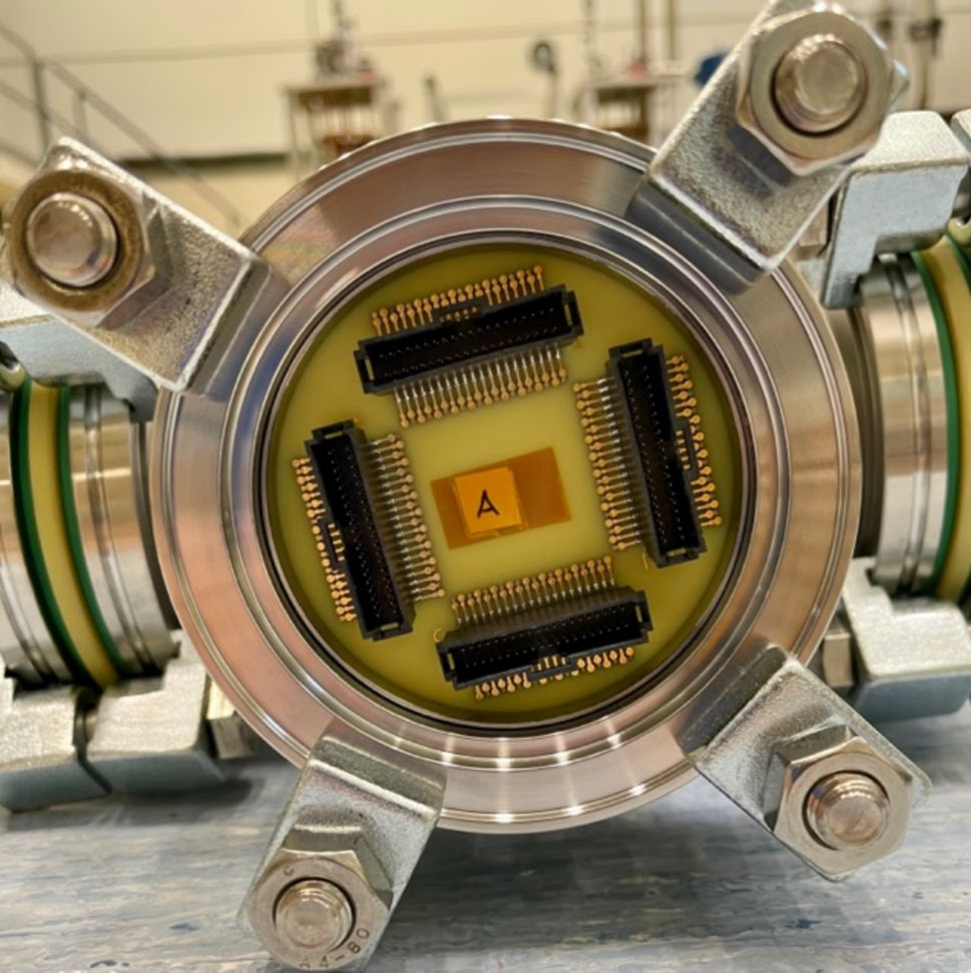
Picture of one 4-mm-thick feedthrough PCB that interfaces the thermometers inside the cryostat and the data acquisition system on the air side.

On the top plate of the cryostat, three 4-mm-thick PCBs are used as feedthroughs to interface the thermometers inside the cryostat and the data acquisition system on the air side. The helium leak rate of these feedthrough PCBs is lower than 10^–8^ mbar l/s. Figure 6 shows one of the feedthrough PCBs.

On both sides of each feedthrough PCB, eight 40-pin insulation-displacement-contact (IDC) connectors are soldered, as shown in Fig. 6. The thermometer boards are connected to the IDC connectors on the vacuum side of feedthrough PCBs by twelve flexible CICOL 30 AWG Micro IDC cables. The additional heat load in the He bath due to those cables is evaluated as lower than 1 W. On the air side of feedthrough PCBs, the electrical signals from thermometers are transmitted to the data acquisition system by twelve standard shielded jacketed ribbon cables with 34 conductors each. Cables inside the cryostat are 3 m long, while those outside the cryostat are 7 m long. The number of conductors in ribbon cables and feedthrough PCBs is intentionally oversized to have enough redundancy in the system.

### Data acquisition

The data acquisition system consists of twelve PCBs that interface the 192 thermometers with six multiplexer switch modules (National Instruments PXIe-2527). After multiplexing, the analog signals from thermometers are digitized by six analog-digital converter modules (National Instruments NI-9251).

Each thermometer is connected to the interface PCBs with two 150 k$\Omega$ resistors in series. The thermometers are all connected in parallel, and a Keithley 2401 voltage source provides the power supply. The voltage difference is usually set to 3 V to supply a ~ 10 µA current for each thermometer. The voltage source is connected to a computer by a general-purpose interface bus to remotely control the power supply. To avoid issues concerning the self-heating of thermometers, a current value lower than ~ 25 µA is recommended^22,23^.

The 32-channel 2-wire multiplexer switch modules are used for multiplexing the analog signals of thermometers. Each module is capable of multiplexing 32 differential inputs. The output of each multiplexer is connected to one channel of a 24-bit analog-digital converter (ADC) module to digitize the voltage drop of each thermometer, connected in input to the multiplexer switch module. Analog signals can be digitized by these ADC channels in the range between −3 V and +3 V with high resolution. The typical voltage drop of thermometers is expected to be between ~ 10 mV and ~ 70 mV at liquid He temperatures. In addition, the ADC channels are equipped with mini-XLR cables to minimize noise during the data acquisition. Unlike the 192 thermometers, the four calibrated thermometers are directly connected to two ADC modules bypassing the multiplexing stage, while the power supply is provided by a Keithley 2401 voltage source, which is only dedicated to these four carbon ceramic thermometers. The acquisition time of each thermometer response and the sampling rate of ADCs can be opportunely tuned to map the temperature of the whole cavity with satisfactory accuracy. The maximum sampling rate of ADCs is 100 kS/s. Due to the high sampling rate of ADCs, high-speed temperature mapping with a maximum of twelve thermometers can also be performed by bypassing the multiplexing stage. High-speed temperature mapping allows us to study the dynamic behavior of heat losses.

The data acquisition system is approximately 3 m from the cryostat outside the radiation shielding. Therefore, multiplexers and ADC modules are protected from potential radiation damage.

## RF performance of the cavity

A Nb/Cu 1.3 GHz TESLA-type cavity was tested in a vertical cryostat at different temperatures and accelerating fields. To evaluate the RF losses of the cavity under test, the main observable is the cavity’s unloaded quality factor $Q_{0}$ as a function of the accelerating electric field $E_{acc}$, measured by standard methods with a phase lock loop circuit. A mobile input coupler is used to maintain the critical coupling of the cavity during the test. The measurement of $Q_{0}$ as a function of $E_{acc}$ is carried out by sweeping the input power while maintaining constant the temperature of the He bath.

$Q_{0}$ values of the cavity are measured at 2.4 K both at saturation pressure and in subcooled condition. In parallel to the evaluation of RF performance, temperature maps are acquired for several values of accelerating field ranging from ~ 2 to ~ 7 MV/m. The heat losses of the cavity became high enough at ~ 2 MV/m to be detected by thermometers, whereas the measurement of $Q_{0}$ is stopped at ~ 7 MV/m in accordance with the safety regulations of the CERN facility where the test has been performed. Indeed, during each $Q_{0}$ scan, the radiation level around the cryostat was continuously monitored, and it exceeded the allowed threshold at ~ 7 MV/m at the onset of field emission processes in the cavity. After filling the cryostat with liquid He at ~ 4.2 K, the temperature of the He bath is decreased by pumping on the He bath until the cryostat pressure reaches 80 mbar, corresponding to a bath temperature of 2.4 K. Using a PID pressure controller, the pressure is always kept constant during the RF cavity test within the experimental uncertainty of 0.2 mbar. The weight of the liquid He causes a vertical temperature stratification in the cryostat, leading to a temperature difference of approximately 17 mK along the cavity cell. The experimental setup also allows us to characterize the cavity at 2.4 K in the subcooled condition. Indeed, when the He bath is at 2.4 K, the cryostat can be quickly re-pressurized to atmospheric pressure. This leaves the liquid He at a lower temperature than that corresponding to its vapor pressure. Due to the overpressure in the cryostat, the activation of nucleation sites on surfaces immersed in the liquid He bath is suppressed and, as a consequence, the nucleate boiling regime is inhibited. Heat transfer by nucleate boiling is more effective than by convection cooling^16^; therefore the cooling capability of the He bath in the subcooled condition is lower than that at saturation pressure^21^.

**Figure 7:**
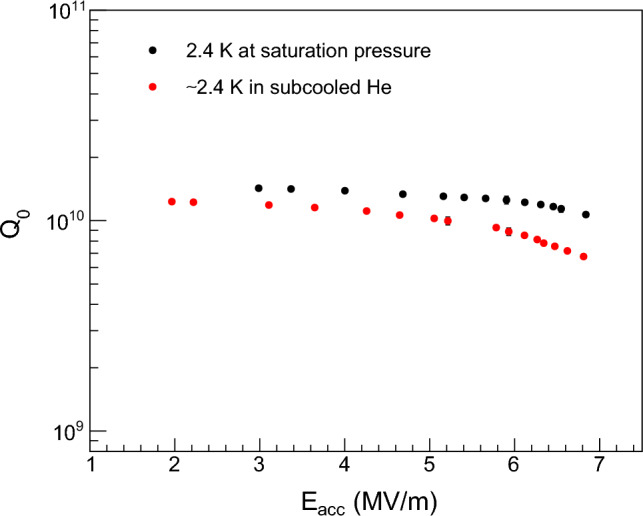
Quality factor $Q_{0}$ of the cavity under test as a function of accelerating electric field $E_{acc}$ at the temperature of 2.4 K at saturation pressure (black) and at ~ 2.4 K in subcooled He (red). Statistical error bars are hidden by markers.

Figure 7 shows the quality factor $Q_{0}$ of the cavity as a function of accelerating electric field $E_{acc}$ at 2.4 K at saturation pressure and in subcooled He. As demonstrated in a previous study^18^, these two He bath conditions ensure satisfactory temperature measurements along a Nb/Cu 1.3 GHz cavity cell. In both cases, progressive performance degradation with the accelerating field is observed in the cavity under test. At 2.4 K at saturation pressure, the $Q_{0}$ decreases by ~ 20% from 3 to 7 MV/m, while the decrease is more than 40% at ~ 2.4 K in subcooled He. In the subcooled condition, the temperature of the He bath is not stable over time; in fact, the temperature slowly drifts to higher values. For this reason, the $Q_{0}$ scan acquired in subcooled He is carried out at approximately 2.4 K.

As shown in Fig. 7, $Q_{0}$ values of the cavity in subcooled He are lower than those at 2.4 K at saturation pressure. This is mainly due to two reasons. Firstly, the $Q_{0}$ scan in subcooled He is taken when the temperature of the He bath is slightly higher than 2.4 K, whereas the $Q_{0}$ at saturation pressure is measured precisely at 2.4 K. The temperature increase of the He bath implies a higher surface resistance of the superconducting film and, in turn, a lower quality factor. Secondly, the heat dissipation from the cavity to the He bath in the subcooled condition is generally lower than when the He bath is at saturation pressure. Because of the limited heat transfer into the He bath, the superconducting film in subcooled He turns out to be warmer than that at saturation pressure for the same accelerating field and temperature of the He bath. As a consequence, the surface resistance of the film increases and the $Q_{0}$ value decreases.

## Temperature maps

**Figure 8:**
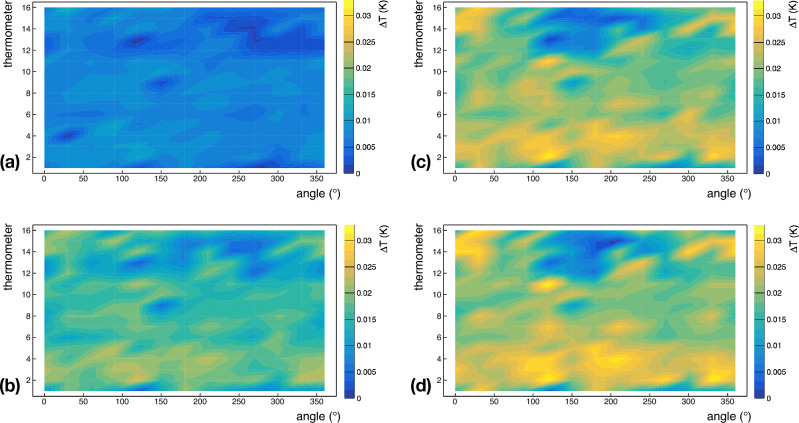
Temperature maps of the Nb/Cu cavity, under test, acquired at 3.0 MV/m (**a**), 4.6 MV/m (**b**), 5.8 MV/m (**c**), and 6.8 MV/m (**d**). Experimental data are taken at 2.4 K when the He bath is at saturation pressure. The abscissa of each plot represents the azimuthal coordinate around the cavity, while the vertical axis of each plot represents the thermometer number. The equator of the cavity cell is between thermometer numbers 8 and 9, whereas the iris at the bottom of the cavity cell corresponds to thermometer number 2, and the one at the top is in correspondence with thermometer number 15.

For evaluating heat losses of the cavity under test, we acquired some temperature maps around the whole surface of the cavity cell at different values of accelerating fields. Figures 8a–d show the temperature maps at 2.4 K at saturation pressure acquired at 3.0 MV/m, 4.6 MV/m, 5.8 MV/m and 6.8 MV/m, respectively. The abscissa of each plot in Fig. 8 represents the azimuthal coordinate around the cavity, while the vertical axis of each plot represents the thermometer number. The equator of the cavity cell is between thermometer numbers 8 and 9, whereas the iris at the bottom of the cavity cell corresponds to thermometer number 2, and the one at the top is in correspondence with thermometer number 15. As expected, the outer surface of the cavity cell is warmer by increasing the accelerating field. Indeed, the average temperature rise of the cavity surface is ~ 8 mK at 3 MV/m, ~ 14 mK at 4.6 MV/m, ~ 16 mK at 5.8 MV/m and ~ 20 mK at 6.8 MV/m.

**Figure 9:**
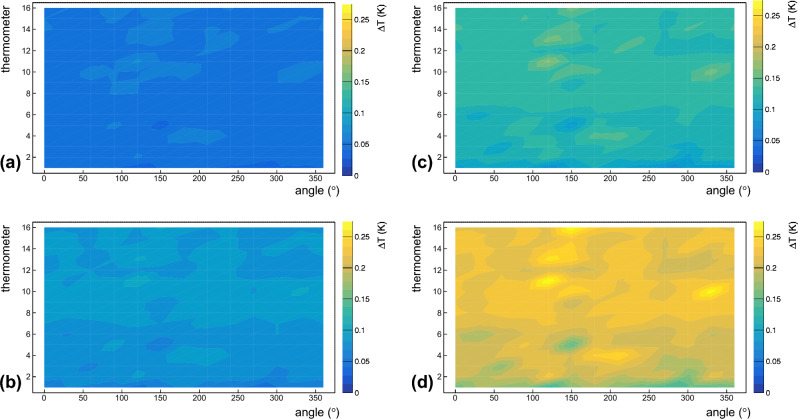
Temperature maps of the Nb/Cu cavity, under test, acquired at 3.0 MV/m (**a**), 4.6 MV/m (**b**), 5.8 MV/m (**c**), and 6.8 MV/m (**d**). Experimental data are taken at ~ 2.4 K when the He bath is in the subcooled condition. The abscissa of each plot represents the azimuthal coordinate around the cavity, while the vertical axis of each plot represents the thermometer number. The equator of the cavity cell is between thermometer numbers 8 and 9, whereas the iris at the bottom of the cavity cell corresponds to thermometer number 2, and the one at the top is in correspondence with thermometer number 15.

Nb/Cu cavities are usually operated in a liquid He bath at saturation pressure. However, for a better characterization of the cavity under test, we also acquired temperature maps at ~ 2.4 K in subcooled He. The heat transfer from the cavity to the He bath in subcooled He is less effective than that at saturation pressure because the cooling mainly occurs by convection. Indeed, the overpressure over the liquid He bath impedes the onset of the nucleate boiling regime. Figures 9a–d show the temperature maps at ~ 2.4 K in the subcooled condition acquired at 3.0 MV/m, 4.6 MV/m, 5.8 MV/m and 6.8 MV/m, respectively. Unlike temperature maps at 2.4 K at saturation pressure, the temperature range of Fig. 9 is about 10 times larger than that for Fig. 8.

## Characterization of heat losses

**Figure 10:**
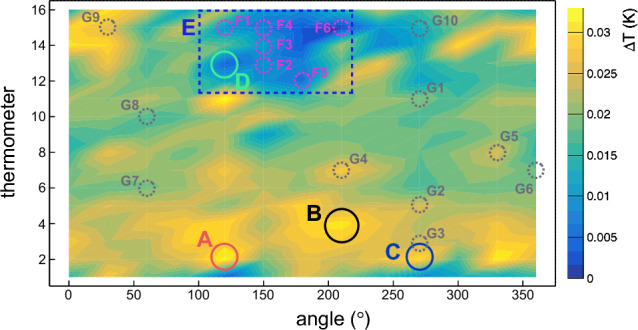
Temperature map of the Nb/Cu cavity under test, acquired at ~ 6.8 MV/m with a $Q_{0}$ value of ~ 1.1 × 10^10^ while the He bath was at 2.4 K at saturation pressure. Specific sites of interest are labeled on the figure, aiming to facilitate their identification throughout the remainder of this study. The abscissa of the plot represents the azimuthal coordinate around the cavity, while the vertical axis of each plot represents the thermometer number. The equator of the cavity cell is between thermometer numbers 8 and 9, whereas the iris at the bottom of the cavity cell corresponds to thermometer number 2, and the one at the top is in correspondence with thermometer number 15.

We characterized the types of heat losses observed in the temperature maps, specifically focusing on those exhibiting behavior consistent with Joule heating or field emission heating. Figure 10 shows the temperature map of the cavity at 2.4 K at saturation pressure, with various sites of interest appropriately labeled to facilitate their identification throughout the remainder of this study. This temperature map was acquired at ~ 6.8 MV/m with a $Q_{0}$ value of ~ 1.1 × 10^10^ while the He bath was at 2.4 K at saturation pressure.

In Fig. 10, different areas of interest are labeled by letters. Hotspots A, B, and C are located in the lower half-cell and represent some of the warmest sites of the cavity during the test. An additional site, labeled by D, shows an interesting behavior, as will be discussed in this section. Sites F1 through F6 delineate the area labeled as E, where an unexpected temperature drop was observed only at saturation pressure, as will be discussed in detail in Section “Nucleate boiling regime”. Finally, sites G1 through G10 are randomly selected across the entire cavity surface for comparison with all the aforementioned sites.

**Figure 11:**
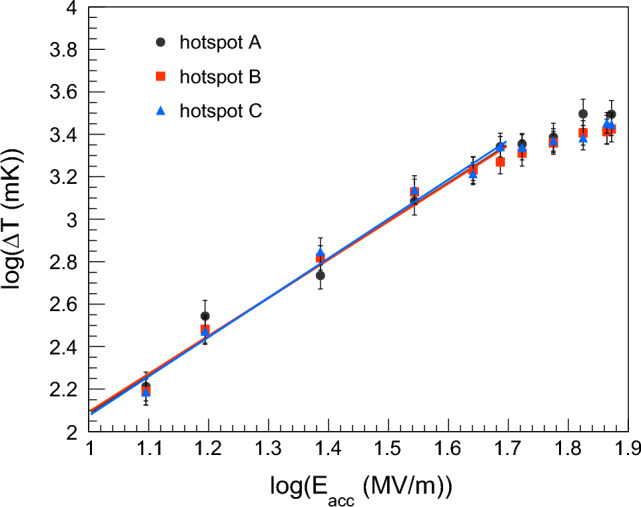
Temperature variation $\Delta T$ as a function of the accelerating electric field $E_{acc}$ in correspondence of hotspots A, B, and C, according to the notation of Fig. 10. Experimental data in the log-log plot are taken at 2.4 K at saturation pressure. Straight lines interpolate data for values of $E_{acc}$ lower than ~ 5.5 MV/m. Markers hide some statistical error bars.

If we assume that the heat transfer from the cavity to the He bath and the surface resistance of the superconducting film are uniform, the temperature rise measured close to a heat loss with ohmic behavior scales as the square of accelerating electric field $E_{acc}$^24^. Figure 11 shows the temperature variation $\Delta T$ as a function of $E_{acc}$ in a log-log plot in correspondence of hotspots A, B, and C, according to the notation of Fig. 10.

By interpolating the experimental data, we found that the slope of lines in Fig. 11 is 1.81 ± 0.12 for hotspot A, 1.79 ± 0.12 for hotspot B and 1.85 ± 0.11 for the hotspot C when $E_{acc}$ is lower than ~ 5.5 MV/m. If the measured temperature rise is due purely to Joule heating, the slope is equal to 2^24^. The statistical z-test confirms that all three lines that interpolate the data in Fig. 11 have a slope compatible with the expected value of 2. Therefore, the behavior of hotspots A, B, and C is ohmic for $E_{acc}$ values lower than ~ 5.5 MV/m. For values of $E_{acc}$ higher than ~ 5.5 MV/m, the temperature rise in all three hotspots does not depend on the square of the accelerating electric field anymore. This can be correlated to the temperature drop observed in area E, as discussed in Section “Nucleate boiling regime”.

**Figure 12:**
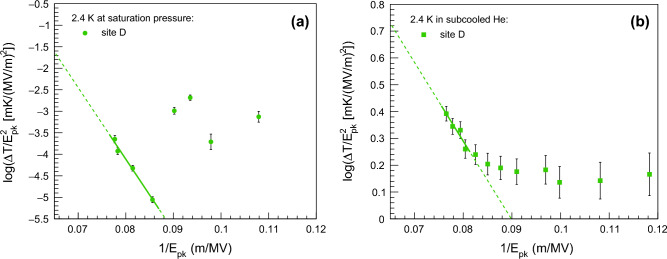
Fowler-Nordheim plots in correspondence of site D, according to the notation of Fig. 10. In Fowler-Nordheim plots, ln($\Delta T$/$E_{pk}^{2}$) is plotted as a function of 1/$E_{pk}$, where $\Delta T$ is the temperature variation and $E_{pk}$ is the peak electric field. Experimental data are acquired at 2.4 K at saturation pressure (**a**) and in subcooled He (**b**). Data interpolation is done by solid straight lines for values of $E_{pk}$ greater than ~ 11.6 MV/m in (**a**) and ~ 12.3 MV/m in (**b**), while the dashed lines in both (**a**) and (**b**) represent the extrapolation of the lines of best fit.

Temperature measurements of the cavity surface can localize hotspots exhibiting behavior consistent with field emission heating. This is accomplished by using Fowler–Nordheim plots, where ln($\Delta T$/$E_{pk}^{2}$) is plotted against the inverse of the peak electric field (1/$E_{pk}$)^24^. For the tested cavity, the ratio between the peak electric field $E_{pk}$ and the accelerating electric field $E_{acc}$ is 1.98^25^. Figures 12a and b show the Fowler-Nordheim plots in correspondence with site D at 2.4 K at saturation pressure and in the subcooled condition, respectively. In the Fowler-Nordheim plot of Fig. 12a, the linear trend for $E_{pk}$ values greater than $\sim$11.6 MV/m suggests that site D exhibits behavior consistent with field emission heating. This is further supported in Fig. 12b, where it is possible to clearly observe the kink caused by field emission heating. The slope of the line that interpolates data in Fig. 12a can be used to determine the effective emission area. A complete description of field emission in SRF cavities and Fowler-Nordheim plots is beyond the scope of this study.

### Nucleate boiling regime

**Figure 13:**
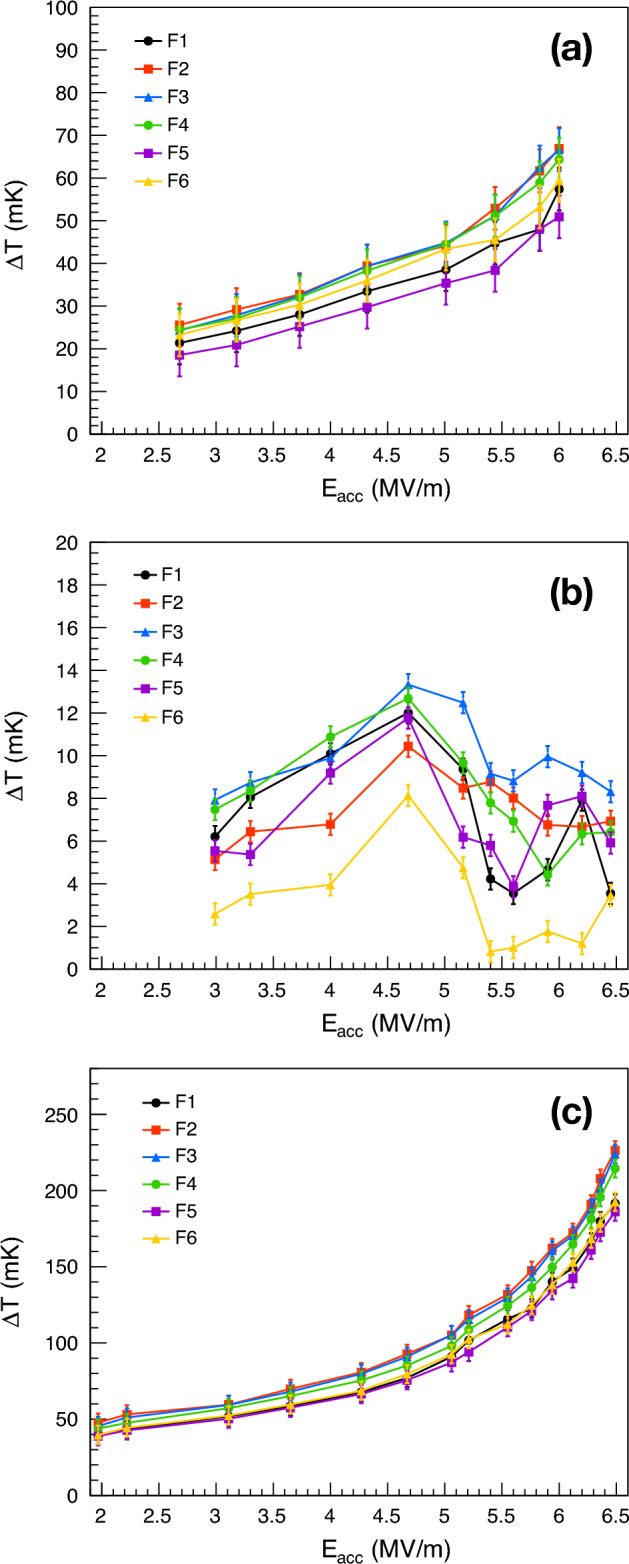
Temperature variations $\Delta T$ measured at sites F1 through F6 with the He bath at ~ 2.2 K in the subcooled condition (**a**), at 2.4 K at saturation pressure (**b**), and at ~ 2.4 K in the subcooled condition (**c**). The temperature range in the vertical axis changes in all three plots.

Systematic acquisitions of temperature maps at different temperatures of the He bath, both at saturation pressure and in the subcooled condition, were carried out to better investigate area E. Temperature measurements were sequentially acquired by changing only the He bath conditions. Figure 13 shows the temperature measurements taken at sites F1 through F6, first with the He bath at ~ 2.2 K in the subcooled condition (a), then at 2.4 K at saturation pressure (b), and finally at ~ 2.4 K in the subcooled condition (c). The temperature of the He bath in subcooled conditions has been intentionally chosen at temperatures lower and higher than that at saturation pressure. In contrast to the observed increasing trend of temperature variation $\Delta T$ with the accelerating field in subcooled He, a temperature drop is measured at around 4.7 MV/m when the He bath is at saturation pressure. Sites F1 through F6 are conveniently selected to define the entirety of area E, however, this temperature drop is observed in most of the sites within area E. Following the temperature drop, the temperature does not appear to increase with the increase of the accelerating field; on the contrary, it seems to remain constant for $E_{acc}$ values higher than ~ 5.5 MV/m, except for small fluctuations.

**Figure 14:**
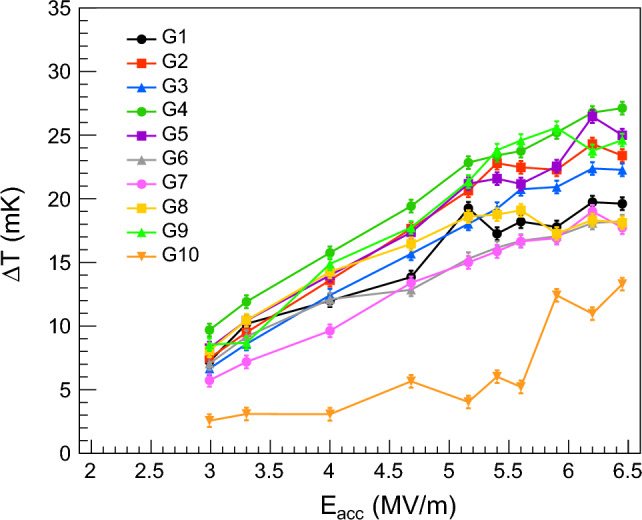
Temperature variation $\Delta T$, measured in ten different sites, as a function of accelerating field $E_{acc}$ at 2.4 K when the He bath is at saturation pressure. The position of sites G1 through G10 is randomly chosen across the cavity surface and indicated in Fig. 10.

Contrary to the temperature drop measured in most of the sites within area E, the temperature across the rest of the cavity surface increases with the increase of the accelerating electric field. This is demonstrated in Fig. 14, where the temperature variation is measured as a function of the accelerating field in ten different sites, randomly chosen and indicated in Fig. 10. In all these sites, which are equally distributed in the upper and lower half-cells, the temperature increases with the increase of the accelerating field, except for small fluctuations. Most of the sites shown in Fig. 14 exhibit a behavior consistent with Joule heating for accelerating field values lower than ~ 5.5 MV/m. Temperature variations in Fig. 14 are measured at 2.4 K when the He bath is at saturation pressure.

Since the temperature drop is observed in most of the sites within area E, the heat in this area might be dissipated by nucleate boiling. Indeed, the transition from convection cooling to nucleate boiling is associated with a temperature drop due to the activation of nucleation sites^16^. In addition, this transition cannot occur when the He bath is in the subcooled condition because the nucleate boiling regime is impeded by the overpressure over the He bath. This provides further evidence to support our hypothesis because we observed the temperature drop only at saturation pressure. For these reasons, our findings suggest that the cavity surface is cooled by convection except for one portion where the transition from convection cooling to nucleate boiling might have occurred.

## Optical inspection of the cavity

**Figure 15:**
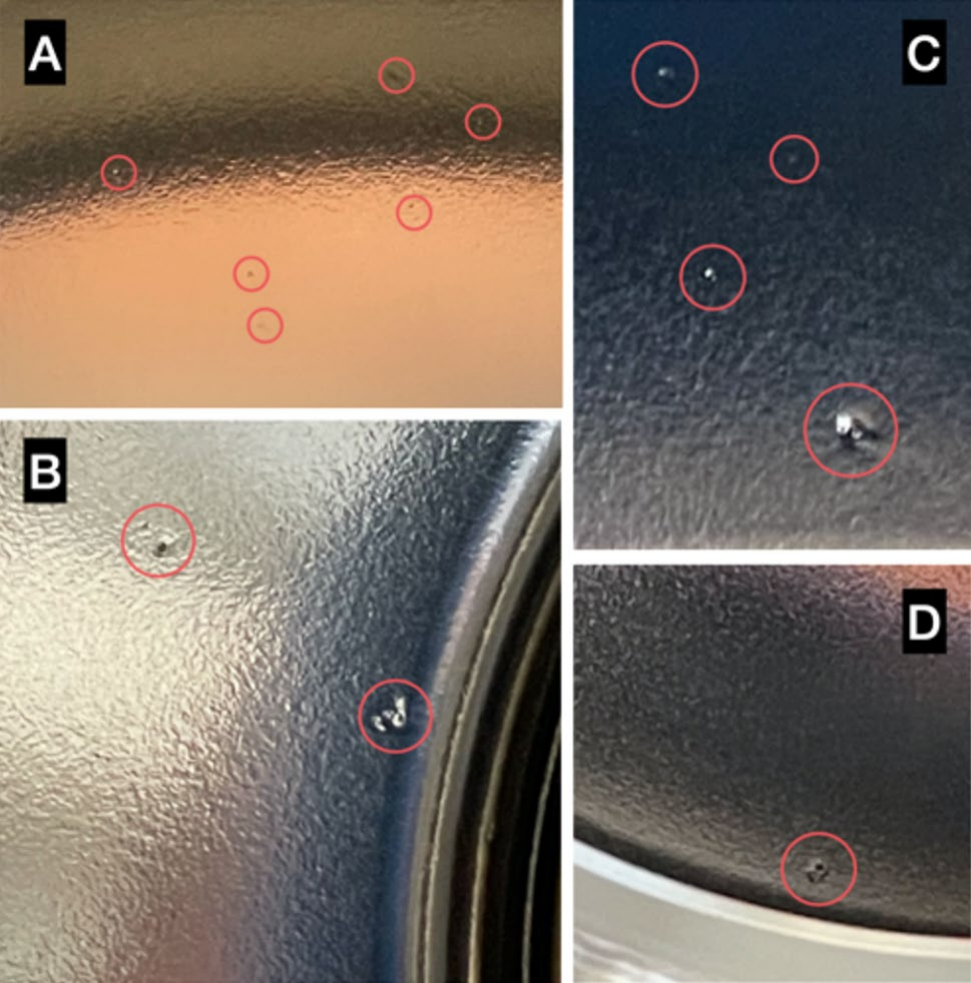
Defects of the Nb thin film in correspondence of sites A, B, C, and D, according to the notation of Fig. 10.

Through an optical inspection of the inner cavity surface, we observed several defects of the Nb film in correspondence with hotspots detected in the temperature maps. We carried out the optical inspection using a commercial camera. Only a limited portion of the inner surface was accessible during the inspection; in fact, we intentionally avoided inserting the camera inside the cavity to avoid damaging it and, if necessary, retesting it for further investigations. Figures 15a–c show the defects in the Nb film in correspondence with hotspots A, B, and C, respectively. These sites display characteristics consistent with Joule heating, as demonstrated in section “Characterization of heat losses”. Figure 15d displays an additional defect observed in correspondence to site D, which shows a behavior consistent with field emission heating. No significant differences are visually observed between the defects with ohmic behavior in Figs. 15a–c, and the defect where behavior consistent with field emission heating was observed.

**Figure 16:**
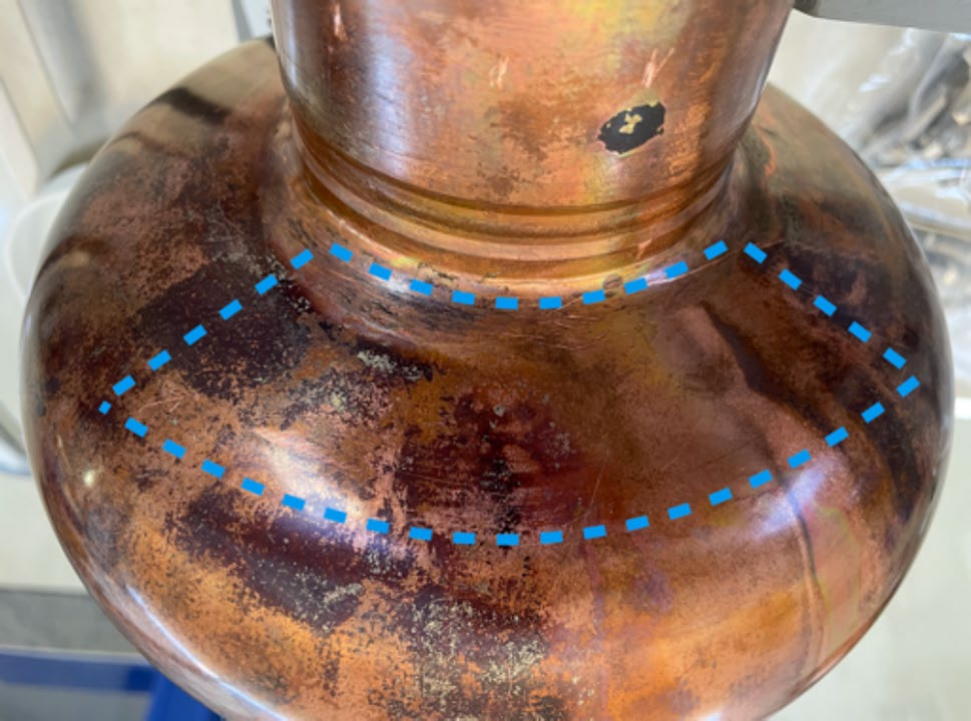
Picture of the outer surface of the Nb/Cu cavity in correspondence with area E, which is surrounded by a dashed curve in blue.

Figure 16 shows the outer surface of the cavity in correspondence with area E. This area is surrounded by a dashed curve in blue. No significant differences between the area E and the remaining cavity surface are observed by optical inspection.

## Heat dissipation of niobium-coated copper cavities

Using our temperature mapping system, we observed that the heat dissipation of the Nb/Cu cavity under test is not uniform over the whole surface at operating conditions. Temperature maps in Fig. 8 clearly show hotspots exhibiting a behavior consistent with Joule heating. In addition, several hints suggest that area E, located in the upper half-cell, is in nucleate boiling regime, as discussed in Section “Nucleate boiling regime”. To dissipate the heat of the cavity surface into the He bath, cooling by nucleate boiling turns out to be more effective than convection. This finding might suggest a way to improve RF performance for Nb/Cu cavities.

Under normal operating conditions, the surface resistance of the superconducting Nb film increases exponentially with temperature^26^. Therefore, limiting the temperature rise of the superconducting Nb films during the operation of SRF cavities implies higher RF performance. For a constant heat flux, enhanced heat transfer into the He bath implies a lower temperature of the RF surface in Nb/Cu cavities that are usually operated in He-I between 4.0 K and 4.5 K at saturation pressure, where the thermal contribution in the surface resistance is relatively high.

A number of authors observed that the heat transfer from Cu surfaces to liquid He-I is generally affected by several factors^16,27,28^. One of those parameters is the roughness of the Cu surface. According to data in the literature^16,18,27,28^, the heat transfer into He-I bath is higher in rough Cu surfaces, where more heat can be dissipated, compared to smooth surfaces.

If the cooling by nucleate boiling regime can be induced in large areas of Nb/Cu cavities by increasing their external roughness, the heat exchange from Nb/Cu cavities to the He-I bath may be enhanced and, in turn, their RF performance improved. Increasing the roughness of the outer surface of Nb/Cu cavities can be achieved by several manufacturing processes. For example, sandblasting could be a straightforward and cheap process that can provide uniform and reproducible results in terms of roughness in large Cu surfaces.

## Conclusions

In this paper, we have described a temperature mapping system based on contact thermometry and designed to effectively sense heat losses in Nb/Cu 1.3 GHz single-cell TESLA-type cavities in He-I both at saturation pressure and in subcooled conditions. Temperature maps in He-I at saturation pressure are acquired to study the heat dissipation from the cavity to the He bath at operating conditions, whereas temperature maps in subcooled conditions allow the localization of hotspots with high precision and accuracy.

Temperature mapping of a 1.3 GHz Nb-coated cavity indicates that the heat dissipation is not uniform over the whole external cavity surface. Indeed, heat losses are localized and can have behavior consistent with Joule heating or field emission heating. In addition, our findings suggest that the cavity under test is mainly cooled by convection cooling, except for one region that is in the nucleate boiling regime.

## Data Availability

The datasets generated during and/or analysed during the current study are available from the corresponding author on reasonable request.
